# Ontogeny of Innate T Lymphocytes – Some Innate Lymphocytes are More Innate than Others

**DOI:** 10.3389/fimmu.2014.00486

**Published:** 2014-10-10

**Authors:** David Vermijlen, Immo Prinz

**Affiliations:** ^1^Faculty of Pharmacy, Université Libre de Bruxelles (ULB), Bruxelles, Belgium; ^2^Institute of Immunology, Hannover Medical School, Hannover, Germany

**Keywords:** T cell, gammadelta T cells, ILC, fetal, neonatal, HSCT, BTN3A1, Skint1

## Abstract

Innate lymphocytes have recently received a lot of attention. However, there are different ideas about the definition of what is “innate” in lymphocytes. Lymphocytes without V(D)J-rearranged antigen receptors are now termed innate lymphoid cells (ILCs) and include cells formerly known as natural killer (NK) cells. Also, lymphocytes that are innate should be able to recognize microbial or stress-induced patterns and react rapidly without prior sensitization, as opposed to adaptive immune responses. Formally, genuine innate lymphocytes would be present before or at birth. Here, we review the ontogeny of human and mouse innate T lymphocyte populations. We focus on γδ T cells, which are prototype lymphocytes that often use their V(D)J rearrangement machinery to generate genetically encoded predetermined recombinations of antigen receptors. We make parallels between the development of γδ T cells with that of innate αβ T cells [invariant (i)NKT and mucosa-associated invariant T cells] and compare this with the ontogeny of innate B cells and ILCs (including NK cells). We conclude that some subsets are more innate than others, i.e., innate lymphocytes that are made primarily early *in utero* during gestation while others are made after birth. In practice, a ranking of innateness by ontogeny has implications for the reconstitution of innate lymphocyte subsets after hematopoietic stem cell transplantation.

## Introduction

Immune responses are traditionally classified into two types: innate and adaptive. In textbooks, immune cells are usually assigned to one of these arms of the immune system, for example, neutrophils and macrophages in the innate arm and conventional T cells, restricted by MHC molecules, in the adaptive arm. Innate lymphocytes, however, can rearrange clonal antigen receptor loci [T cell antigen receptor (TCR) or B cell antigen receptor (BCR)] but at the same time show characteristics of the innate immune system: recognition of molecular patterns, rapid response, and no need for clonal expansion. In addition, there are lymphocytes not expressing a TCR or BCR, including the natural killer (NK) cells with “NK activity” that were discovered 40 years ago (1, 2). More recently, the family of non-T/non-B cells has been extended to include a series of other innate lymphocytes collectively called “innate lymphoid cells (ILCs)” (3). However, the boundaries between lymphocytes of the innate and adaptive immune system are blurred. On the one hand, non-T/non-B innate lymphocytes such as NK cells can adapt to their environment (4) and even possess memory characteristics after viral infection (5–7), reviewed in Ref. (8–10). On the other hand, T and B lymphocytes that undergo V(D)J recombination to rearrange their antigen receptor loci can be innate lymphocytes as well: within T cells one has αβ TCR-expressing T cells of which the TCR does not recognize MHC/peptide complexes [invariant NKT (iNKT) cells and mucosa-associated invariant T cells (MAIT) (11, 12) and T cells expressing γδ TCRs (13, 14)], within B cells one has B1 and marginal zone (MZ) B lymphocytes (15, 16).

Literally, “innate lymphocytes” would implicate that these cells are generated before birth and that their presence would not depend on environmental cues. In this review, emphasis will be given to the ontogeny of the different types of innate lymphocytes in humans and mice. In both organisms, some subsets of innate lymphocytes are generated mainly during fetal life, while others are made later in life. The timing of innate lymphocyte generation could depend on the stem cells of which they are derived from and/or the specific fetal environment. This knowledge can be important in clinical settings, for example, to have insight into the reconstitution of innate-like lymphocytes during hematopoietic stem cell transplantation (HSCT).

## Innate-Like T Lymphocytes

### γδ T cells

Like conventional αβ T cells and B cells, γδ T cells use V(D)J gene rearrangement with the potential to generate a set of highly diverse receptors to recognize antigens. This diversity is mainly generated in the complementary-determining region 3 (CDR3) encoded within the TCR or BCR loci (14, 17). The tripartite subdivision of lymphocytes possessing rearranged receptors into B cells, αβ T cells, and γδ T cells has been conserved since the emergence of jawed vertebrates, more than 450 million years ago (18), and may even originate from earlier evolutionary events (19). A major difference between αβ T cells and γδ T cells is the way they recognize antigens. In contrast to conventional αβ T cells, γδ T cells are not dependent on classical MHC molecules presenting peptides. Based on the ligands that have been identified, it appears that γδ TCRs can recognize antigens in an antibody-like fashion, while the TCR of other γδ T cell subsets can bind to the MHC-like protein CD1d loaded with lipids (13, 20–25). Although there are common characteristics among γδ T cells, it is clear that γδ T cells, analogous to αβ T cells, do not represent a homogenous population of cells with a single physiological role (26). γδ T cells are typically grouped according to the type of Vγ chain (in mice) or Vδ chain (in humans) they express. Of note, their TCR recombination machinery is often used to generate always the same γδ TCRs of rather limited diversity and the expression of the respective invariant γδ TCR recombinations is often associated with their anatomical location and/or function (14, 27, 28).

#### Human γδ T cells

**Figure 1:**
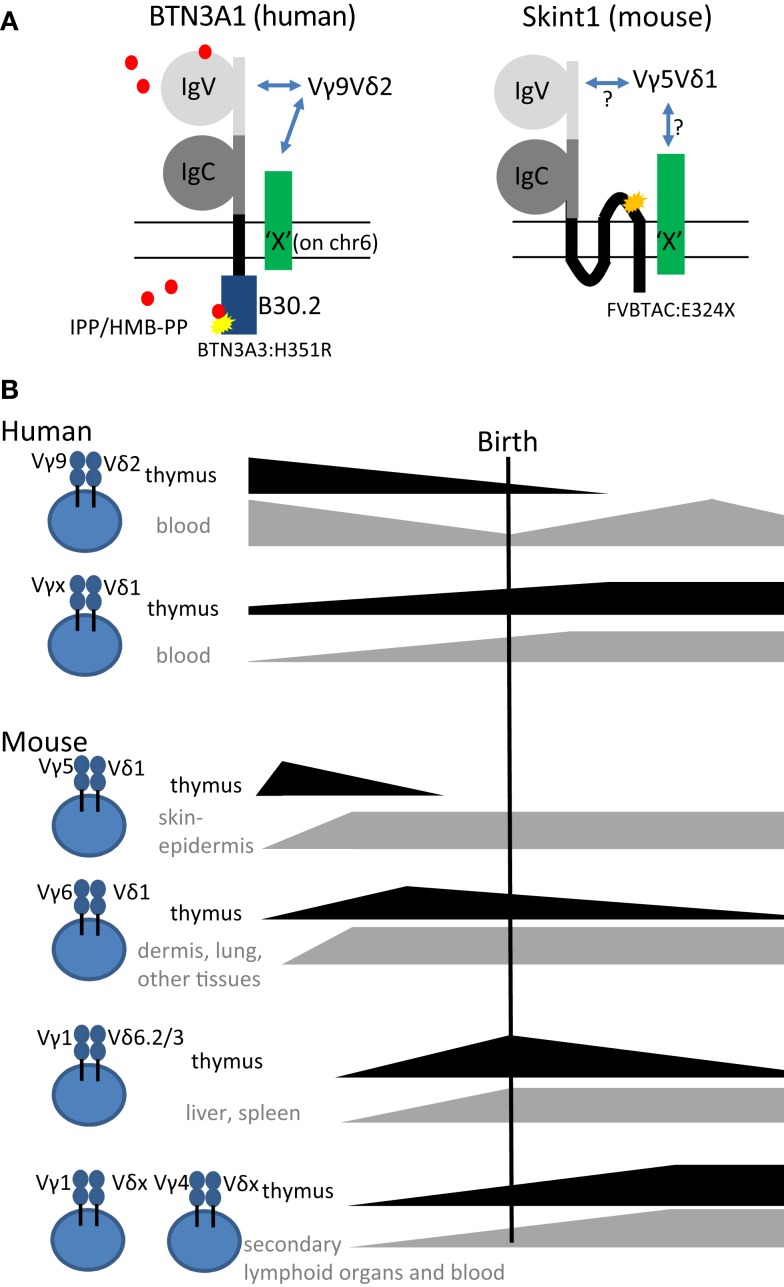
**(A)** Schematic representation of the structures of the super immunoglobulin family members BTN3A1 (human) and Skint1 (mouse) (possibly) implicated in the selection of human Vγ9Vδ2 and mouse Vγ5Vδ1 T cells, respectively. Note the three transmembrane domains in Skint1, while there is only one for BTN3A1. Both for BTN3A1 and for Skint1, interaction partners have been suggested to exist (illustrated by the green molecule “X”), that could be the actual ligands of the Vγ9Vδ2 and Vγ5Vδ1 TCR. The yellow star in BTN3A1 illustrates the one amino acid difference with the BTN3A3 B30.2 (histidine instead of arginine at position 351). BTN3A2, the third member of the BTN3A subfamily, lacks completely the intracellular domain B30.2. The orange star in Skint1 illustrates a spontaneous mutation found in FVB mice from Taconic changing the glutamate toward a stop codon at amino acid position 325. Phosphoantigens (IPP/HMB-PP, red dots) are proposed to be presented extracellularly by the IgV domain or sensed intracellularly by the B30.2 domain. **(B)** Illustration of thymic production, persistence, and export of human and mouse γδ T cell subsets before, around, and after birth.

In humans, the two prevailing subsets of γδ T cells are characterized as Vδ2-positive (Vδ2^+^) and Vδ2-negative (Vδ2^–^) γδ T cells. The former expresses the TCR variable (V) region pair Vγ9Vδ2 (main subset in adult peripheral blood) and the latter expresses Vδ1 paired with various Vγ elements (enriched in tissues such as adult gut and liver). The Vγ9Vδ2 subset has been shown to react specifically toward non-peptide low molecular weight phosphorylated metabolites (so-called phosphoantigens) derived from the isoprenoid metabolic pathway (29). These phosphoantigens can be microbial-derived – (E)-4-hydroxy-3-methyl-but-2-enyl pyrophosphate (HMB-PP), derived from the microbe-specific 2-C-methyl-d-erythritol 4-phosphate (MEP) pathway of isoprenoid metabolism is the most potent phosphoantigen described- or host-derived such as isopentenyl pyrophosphate (IPP) (29). Recently, the butyrophilin BTN3A1 has been shown to be involved in sensing or presentation of these phosphoantigens (30–32) (Figure 1A). Butyrophilins belong to the immunoglobulin superfamily, and the subfamily BTN3A contains three different members: BTN3A1, BTN3A2, and BTN3A3 (30–33). Each subfamily member contains an extracellular N-terminal IgV and a membrane-proximal IgC domain connected to a single-pass transmembrane domain. BTN3A1 and BTN3A3 both contain an intracellular B30.2 domain, which is missing in BTN3A2 (Figure 1A). As recently discussed at the 6th International Gamma Delta T-Cell Conference in Chicago (May 16–18, 2014), it is not conclusively established how binding of phosphoantigen induces allosteric conformational changes of BTN3A1 and how this leads to activation of Vγ9Vδ2 T cells. However, it is conceivable that additional molecules are involved (34) (represented by “X” in Figure 1A). While phosphoantigen-reactive Vγ9Vδ2 T cells are thought to be restricted to primates, there is recent evidence that Vγ9, Vδ2, and BTN3A1 genes are co-conserved across a variety of vertebrate species including primates, alpaca, armadillo, sloth, dolphin, dromedary, orca, but not rodents (35).

Although human circulating T cells can be detected as early as 12.5 weeks of gestation, most information on T cells in early life, including γδ T cells, is derived from studies on cord blood at term delivery (>37 weeks of gestation) (36). In contrast to adult peripheral blood γδ T cells, human neonatal cord blood γδ T cells express diverse Vγ and Vδ chains paired in a variety of combinations (37, 38). Thus, the adult-like Vγ9Vδ2 subpopulation only represents a small fraction of the neonatal γδ T cells at term delivery; the major fraction are rather Vγ9^–^Vδ1^+^ cells (37–41). The current picture is that the predominance of the Vγ9Vδ2 subset in adult blood is due to post-natal expansion of cells expressing particular CDR3 in response to encounter with microbes, especially those generating phosphoantigens derived from the microbe-specific MEP pathway of isoprenoid synthesis (27, 39, 42–44) (Figure 1B). Furthermore, this subset has been shown to differentiate rapidly after birth within the first year of life (45). This view is consistent with recent deep sequencing studies that found a limited diversity in the adult *TRG* (T cell receptor gamma) repertoire. There, the *TRG* repertoire of peripheral blood γδ T cells from three independent donors was dominated by canonical Vγ9JγP sequences, which made up to 45% of all amplified *TRG* sequences (46). Cord blood γδ T cells can produce already significant amounts of IFN-γ after a brief polyclonal stimulation (38, 45, 47). Emphasizing the acquisition of functional competence *in utero*, IFN-γ was produced by γδ T cells sampled from premature births, and, although 1 month’s post-partum environmental exposure invariably increased their TNF-α production, it had no consistent effect on IFN-γ (47). To investigate whether the human fetus could produce particular “fetal-type” of γδ T cells, we recently investigated γδ subsets in human fetal blood before birth. Surprisingly, rather than requiring post-natal microbial exposure, Vγ9Vδ2 T cells turned out to be the predominant blood subset in the second trimester fetus and expressed a semi-invariant TCR (48). This population is later on replaced by other γδ T cell subsets so that Vδ1^+^ T cells predominate at birth (Figure 1B). Combined with a low level or absence of the Vδ2 chain in post-natal thymi (39, 49, 50), our observations point toward a fetal wave of blood Vγ9Vδ2 production before 30 weeks of gestation (Figure 1B). Thus, it appears that human Vδ2^+^ T cells, and in particular Vγ9Vδ2 T cells with canonical Vγ9JγP sequences (51), are more innate than Vδ2^−^ (Vδ1^+^ and Vδ3^+^) T cells.

#### Mouse γδ T cells

γδ T cells are the first T cells that leave the thymus in mice. Those primary T cells are mainly innate γδ T cells that use their TCR recombination machinery to generate always the same γδ TCRs with no or little junctional diversity (14, 27). Specifically, the first specialized γδ T cell population, called dendritic epidermal T cells (DETCs), is exclusively generated before birth (Figure 1B). It migrates to and populates the mouse skin epidermis already *in utero*. It has a fixed TCR composed of invariant Vγ5Jγ1Cγ1 and invariant Vδ1Dδ2Jδ2 (Vγ5Vδ1 TCR in short) without P- or N-nucleotides (52, 53). The same canonical Vδ1Dδ2Jδ2 chain is employed in combination with an invariant Vγ6Jγ1Cγ1 TCR chain in IL-17-producing Vγ6Vδ1 T cells. The development of Vγ6Vδ1 cells is also confined to the embryonic thymus (Figure 1B). Vγ6Vδ1 cells were initially thought to be restricted to uterus and tongue (54) but subsequently also found in many other tissues such as lung (55), liver (56), dermis (57, 58), secondary lymphoid organs (59), and intestinal lamina propria (60). Next, IL-4 producing Vγ1^+^ γδ NKT cells with restricted Vδ6Dδ2Jδ1 junctions and semi-invariant Vγ1Jγ4Cγ4 junctions develop perinatally and preferentially localize in liver and spleen (61–63) (Figure 1B). In contrast, γδ T cells circulating in blood and secondary lymphoid organs mostly contain either Vγ1 or Vγ4 rearrangements. These are also produced after birth and are thought to have highly diverse TCR repertoires (27, 28, 54, 64, 65). In particular, the pool of peripheral Vγ4^+^ T cells is heterogeneous and contains both innate IL-17-producing cells and cells that are rather biased to IFN-γ production. These can be segregated according to a CCR6^+^CD27^−^ and CCR6^–^CD27^+^ surface marker phenotype, respectively (66–68). In addition, Vγ4^+^ T cells also comprise CD27^+^CD45RB^high^ cells, a subset that readily produces IFN-γ upon stimulation with IL-18 and IL-12 (69). Furthermore, the requirements for final differentiation of Vγ4^+^ and other T cells into effector γδ T cells may vary between γδ T cell types depending on their ontogeny (70). For example, it was recently proposed that Vγ4^+^ T cells but not Vγ6^+^ T cells require extrathymic environment for imprinting of skin-homing properties and acquisition of an IL-17-producing phenotype (71).

#### Selection and peripheral activation of γδ T cells

The above described characteristics of mouse and human γδ T cells [fetal wave of production, (semi-) invariant TCR, pre-programing for production of IFN-γ or IL-17] have been linked in the mouse to the action of selecting elements, most notably Skint1, an immunoglobulin superfamily member, that selects the murine intraepidermal Vγ5Vδ1 T cell repertoire (13, 66, 72, 73) (Figure 1A). The possibility exists that the butyrophilin BTN3A1 may act as a selecting element for Vγ9Vδ2 T cells (Table 1), given its role in mediating stimulation by phosphoantigens, and its striking homology to Skint1, so far the only known natural selecting element for γδ T cells (13, 30–32, 73–75) (Figure 1A). This would establish a much stronger parallel between human and murine γδ T cells than is usually articulated, as phosphoantigen-reactive Vγ9Vδ2 T cells are described to be restricted to primates. Other parallels between human Vγ9Vδ2 and mouse Vγ5Vδ1 cells are that both the *V*γ*5* (*TRGV5*) and the *V*γ*9* (*TRGV9*) gene segments are the most downstream located (and thus closest to the *C*γ*1* gene segment) within the *TRG* gene cluster (76). In the mouse, this localization has been shown to contribute to early production of Vγ5Vδ1 cells (77). Of all human *V*δ gene segments, *V*δ*2* (*TRVD2*) shows the highest similarity to mouse *V*δ*1* (*TRVD1*): they are the only two members of a separate cluster in a dendogram comparing all *V*δ and *Vα* gene segments of human and mouse (78). Thus, different species may produce different variations of early γδ T cells according to their specific needs (such as phosphoantigen-reactive cells in humans and skin-homing cells in mice), but similar mechanisms may be used to achieve this (Figure 1).

**Table 1:** **Overview of innate T cells with their candidate selecting/education elements and associated activators, preferential timing of production, and their reconstitution after stem cell transplantation**.

	γδ T						iNKT		MAIT	
	Human		Mouse				Human	Mouse	Human	Mouse
(Semi-) invariant TCR	Vγ9JγPCγ1	Vγx	Vγ5Jγ1Cγ1	Vγ6Jγ1Cγ1	Vγ1Jγ4Cγ4	Vγ1+ and	Vα24–JαQ	Vα14–Jα14	Vα7.2–Jα22	Vα19–Jα33
	Vδ2	Vδ1^a^	Vδ1Dδ2Jδ2	Vδ1Dδ2Jδ2	Vδ6Dδ2Jδ1	Vγ4+ cells^a^	Vβ11	Vβ8/Vβ7/Vβ2	Oligoclonal CDR3β	

Selecting/education element	BTN3A1?	?	Skint1	?	?	?	CD1d	CD1d	MR1	MR1
	Butyrophilin		Butyrophilin-like on fetal thymic stroma				MHC class I-like on DP thymocytes	MHC class I-like on DP thymocytes	MHC class I-like on DP thymocytes	MHC class I-like on DP thymocytes

Small molecule activator/self antigen derived from cellular metabolism (in case of MAIT: (commensal) microbial metabolism)	Prenyl-pyrophosphate/isoprenoid metabolite (e.g., IPP)	?	?	?	?	?	Lipids (e.g., β-GlcCer; pLPE)	Lipids (e.g., β-GlcCer; pLPE)	Transitory neo-antigens: 5-OE-RU; 5-OP-RU; microbial vitamin B2 precursor + glyoxal or methylglyoxal	Also transitory neo-antigens?: 5-OE-RU; 5-OP-RU; microbial vitamin B2 precursor + glyoxal or methylglyoxal

Preferentially made before birth	+	−	+	+	±(perinatal)	−	+	−	± (?)	−

Reconstitution after HSCT (with reference)	±Poor (79)	+ (79)	−(80)	−(54)	±Poor (63)	+ (59)	+ (82, 83)	+ (84)	?	?

*^a^These TCR are not regarded as (semi-) invariant TCR. However, this does not exclude that these populations include subsets containing (semi-) invariant TCRs*.

*5-OE-RU, 5-(2-oxoethylideneamino)-6-d-ribitylaminouracil; 5-OP-RU, 5-(2-oxopropylideneamino)-6-d-ribitylaminouracil; BTN3A1, butyrophilin 3A1; HSCT, hematopoietic stem cell transplantation*.

Responses of human γδ T cells in early life infections have been investigated recently. Placental malaria, which can produce phosphoantigens via the MEP pathway, has been shown to alter the Vγ9 repertoire and to slightly increase the percentage of central-memory Vγ9Vδ2 T cells (85). Other γδ T cells subsets, such as a public/invariant Vγ8Vδ1 TCR-expressing subset, have been shown to be involved in responses to congenital CMV infection (38) [see “Immune response to Cytomegalovirus in Early Life” within this Research Topic for further details (Huygens et al. under review)]. Thus, it appears that depending on the type of pathogen infecting the fetus, different types of γδ T cell subsets react.

#### Regeneration of γδ T cells in adults

Of note, recent studies have shown that adult HSCT and umbilical cord blood transplantation (UCBT) result in the appearance of γδ T cells in the blood of these patients (79, 86). These γδ T cells were mainly from a different type than the Vγ9Vδ2 T cell subset, and a major influence of CMV on these non-Vγ9Vδ2 T cells was observed. Vγ9Vδ2 T cells failed to reach the median of the normal range during a 2-year follow-up (79), which might indicate that adult bone-marrow or peripheral blood derived hematopoietic stem cells have only a low capacity in generating innate Vγ9Vδ2 T cells (Table 1). This would need further investigation, as until now it is even not clear to what extent the γδ T cells observed in the circulation upon reconstitution are derived from hematopoietic stem cells or rather from pre-existing γδ T cells in the transplant.

In the mouse model, γδ T cells are efficiently regenerated after HSCT (59, 87). Furthermore, the Indu–*Rag1* mouse model, in which deficient V(D)J recombination can be repaired by inversion of *Rag1* in the adult stage via tamoxifen-induced cre recombinase expression (88), showed that γδ T cells can be restored within 2 weeks in adult mice (59). This includes efficient regeneration of a prominent population of intestinal epithelial Vγ7^+^ T cells (γδ iIELs). However, some γδ T cell populations are more innate than others because they depend on the fetal thymic microenvironment, on fetal stem cells, or both and therefore cannot be regenerated in the adult thymus. These inborn γδ T cells cannot be regenerated in adult mice (Table 1). They include the invariant Vγ5Vδ1 cells (DETCs) (80) and IL-17-producing invariant Vγ6Vδ1 T cells (54) as well as natural IL-17-producing γδ T cells with TCRs composed of other Vγ segments (70). In future deep sequencing studies, it is likely that additional public, innate, invariant γδ T cell populations will be discovered.

### Invariant αβ TCR-Expressing T cells: iNKT and MAIT

The vast majority of αβ T cells are conventional T cells, i.e., their TCR recognizes peptides derived from pathogen-derived proteins in the context of MHC class I or II and their TCR repertoire is polyclonal. However, there exist also small subpopulations of innate-like αβ TCR-expressing T cells that are not MHC-restricted and express (semi-) invariant TCRs: iNKT cells and mucosal-associated invariant T (MAIT) cells.

#### iNK T cells

Invariant NKT cells express a semi-invariant TCR that recognizes lipids presented by CD1d, a MHC class I-like molecule, both in human and mice (11). In human, this TCR consists of an invariant Vα24–Jα18 chain that preferentially combines with Vβ11 and the ortholog in mouse is an invariant Vα14–Jα18 that combines with Vβ8/Vβ7/Vβ2. Thus, in contrast to the main γδ T cell population in human adult peripheral blood, namely the primate specific Vγ9Vδ2 T cells described above, CD1d-lipid reactive iNKT cells are conserved both in human and mice. Of note, while the representation of iNKT cells in human blood is highly variable (range from 0.001% till more than 1%), it is clear that in mice iNKT cells are at least 10 times more frequent (11).

The first iNKT cell ligand identified was an α-branched galactosylceramide (abbreviated as the commonly used term αGalCer), a lipid compound extracted from the marine sponge *Agelas mauritianus*. Later, closely related glycosphingolipids that substitute for lipopolysaccharide (LPS) in the cell wall of *Sphingomonas*, a Gram-negative, LPS-negative member of the class of α-proteobacteria, have been identified as iNKT TCR ligands (11). Because it is known that extracts from *A. mauritianus* have different properties depending on season and location and because these sponges are often colonized by α-proteobacterial symbionts, particularly by *Sphingomonas*, the marine sponge αGalCer may in fact have originated from bacterial symbionts (11). Also in protozoa and fungi, iNKT ligands have been identified (89). Thus, like phosphoantigens for Vγ9Vδ2 T cells, exogenous iNKT activators can be found in a variety of pathogens.

More recently, several reports have been published related to the identity of self-antigens for mouse and/or human iNKT cells, which has generated some controversy. These self-antigens could play an important role during the development of iNKT cells, in the (fetal) thymus or periphery. The following molecules have been described to be endogenous iNKT ligands: isoglobotrihexosylceramide (iGb3) (90), lysophosphatidylcholine (LPC) and lysosphingomelin (LSM) (91), the peroxisome-derived ether-bonded phosphoethanolamine compounds eLPA and pLPE (92), and β-glucosylceramide (β-GluCer) (93) (Table 1). Note that iGb3 is unlikely to play an important role for human iNKT cells as humans do not express the relevant synthase and are thus unable to synthesize iGb3 (94). Thus, it is possible that multiple endogenous iNKT antigens exist in human and mouse, which can be important during development in the thymus and/or later in the periphery during infection (93) (Table 1). Thus, as for Vγ9Vδ2 T cells, both exogenous and endogenous/self-derived activators for iNKT cells have been identified.

Whereas iNKT cells are present in the human fetal thymus at higher frequencies at the start of the second trimester, their representation gradually declines with gestational age, and they are present at very low frequency in the post-natal thymus (95, 96). This situation is similar to human Vδ2^+^ T cells and indicates that a relatively large part of the human thymic iNKT cell output occurs during early fetal development. The selecting element CD1d is expressed on thymocytes, and endogenous antigens (β-GlcCer; pLPE) have been described to be expressed in mouse thymus (92, 93) (Table 1). Thus in contrast to the selecting element of the invariant mouse Vγ5Vδ1 T cells (Skint1), which is expressed on fetal stroma (74), iNKT cells are rather selected on CD1d-expressing double-positive (DP) thymocytes (95) (Table 1). Interestingly, it has been recently suggested that distinct mouse iNKT cell subsets (making either IL-4, IFN-γ, or IL-17) may be selected by different selecting self-TCR ligands providing different TCR signal strengths (97), making parallels with the development of IFN-γ versus IL-17-producing γδ T cell subsets (66, 72, 73). iNKT cells can be divided according to their expression of CD4: CD4^+^ and CD4^−^ iNKT subsets. In the human fetal thymus, iNKT cells are largely CD4^+^, and only a small fraction expresses CD161 (95). In contrast to fetal thymus and term cord blood, a majority of the iNKT cells in adult blood lack expression of CD4 and are almost uniformly CD161^+^. Together with change in the expression of other markers, this indicates that iNKT cell differentiation is characterized by gradual loss of CD4 expression and increased expression of CD161 (95). However, it cannot be excluded that the CD4^−^ subset may be a separate lineage that is extrathymically derived or that this subset may develop in the thymus at a later developmental stage (95), and would be thus “less innate” than the CD4^+^ counterpart, though this last possibility is less likely (96). Differences between human and mouse iNKT cell development have been observed: while iNKT progenitors are readily found in human fetal but not in post-natal thymus, progenitors are frequent in adult mouse thymus; and both CD4^+^ and CD4^−^ NK T cell progenitors are found in the mouse thymus, whereas only the CD4^+^ can be detected in the human fetal thymus (95, 98, 99). Thus, it seems that in human, iNKT cells are preferentially made before birth and in mice after birth (Table 1). Coinciding with this is the observation in a mouse tetanus toxoid (TT) immunization model: αGalCer given at the time of primary immunization on post-natal day 17 (“late neonatal stage”) had a significantly higher TT-specific IgM response as well as memory IgG response, while αGalCer given on post-natal day 7 (“early neonatal period”) resulted in only marginal boosting (81). Also, while a high enrichment of murine iNKT cells is observed in the liver (up to 50% of hepatic lymphocytes), no such enrichment is observed in neither the fetal nor adult human liver (100); in human, the highest iNKT frequencies are found in the omentum (101).

In contrast to a previous report (102), it was shown more recently that, despite the fact that human thymus and cord blood iNKT cells in humans were predominantly CD4^+^, they were capable of significant cytokine production after stimulation with PMA/ionomycin, including IFN-γ and TNF-α, illustrating that a large proportion of CD4^+^ NKT cells in thymus and cord blood are functionally competent (103). Also recently, it has been shown that iNKT cells can mature in the second trimester fetus, especially in the fetal intestine, thus before post-natal microbial exposure (100). Indeed, these cells express markers associated with maturation (although percentages of CD4^+^ iNKT cells are relatively high compared to adult iNKT cells) and can readily produce IFN-γ and TNF-α (other cytokines where not investigated) after treatment with αGalCer (100). Despite indications that human iNKT cells are mainly generated in the fetal thymus (95), it appears that iNKT cells are well reconstituted after HSCT and UCBT, with the CD4^+^ subset appearing before the CD4^−^ subset β (82, 83, 104) (Table 1). Murine iNKT cells are reconstituted as well at 12 weeks after HSCT (84) (Table 1).

#### Mucosa-associated invariant T cells

Mucosa-associated invariant T cells, so-called because of their enrichment in gut lamina propria, express a semi-invariant αβ TCR, which recognizes (modified) microbial vitamin B2 (riboflavin) metabolites presented by the MHC class I-like molecule MR1. Like iNKT cells, these cells are conserved in human and mice (12, 105, 106). In humans, this TCR consists of Vα7.2–Jα22 and in mouse of Vα19–Jα33, with two variable amino acids in the CDR3 V–J junction, and recently is has been shown by high-throughput sequencing that the CDR3β repertoire of human MAIT cells is oligoclonal (107). Also recently, it has been demonstrated that the actual antigens bound to MR1 and recognized by the human MAIT TCR are transitory neo-antigens, 5-(2-oxoethylideneamino)-6-d-ribitylaminouracil (5-OE-RU) and 5-(2-oxopropylideneamino)-6-d-ribitylaminouracil (5-OP-RU), formed by a non-enzymatic reaction of the riboflavin metabolite 5-amino-6-d-ribitylaminouracil (5-A-RU) with small molecules, such as glyoxal and methylglyoxal, which are derived from other metabolic pathways (108) (Table 1). It is not clear whether endogenous/self-antigens exist that are similar to these microbial-derived antigens, as is the case for Vγ9Vδ2 T cells and iNKT cells, and that could play a role in the selection of MAIT cells (109). Although MAIT cells appear to be relatively rare in mice, they constitute a very significant population of T cells in humans, accounting for between 1 and 10% of T cells in peripheral blood. Thus, on this aspect MAIT cells appear to be the counterpart from the iNKT cells: MAIT are high in human and low in mice, while for iNKT, the opposite scenario is the case.

In both mouse and humans, it is generally accepted that MAIT cells develop in the thymus (105, 110, 111). Based on data using TCR-transgenic mice, it was shown that MAIT cells can be selected by MR1 expressed on DP thymocytes (112). This is similar to iNKT cells (CD1d) (112), but different from the invariant Vγ5Vδ1 T cells (Skint1) where the selecting element is expressed on the thymic stroma (74) (Table 1). Human MR1 also has been detected primarily on CD3^+^ thymocytes (113), suggesting that human MAIT cells might also be selected by DP thymocytes, similar to what was described for mouse MAIT cells (112). Human MAIT cells are rare and immature in the fetal thymus, spleen, and mesenteric lymph nodes (111). However, maturation (such as the capacity to produce IFN-γ) can take place in fetal mucosal tissues (most pronounced in the intestine), thus before post-natal microbial exposure (111). While cord blood MAIT cells show a naïve phenotype, they do show effector capacity (TNF-α production) in response to *Mycobacterium tuberculosis*-infected cells (113). MAIT cells from adult peripheral blood on the other hand show a memory phenotype and have undergone substantial peripheral expansion, thus indicating post-natal response after antigen exposure, possibly from bacterial flora (110, 113). This is consistent with the fact that the accumulation of mouse MAIT cells in the gut lamina propria requires the presence of microbiota (106). This contrasts with iNKT cells, where their accumulation in tissues is not changed in germ-free mice (114).

## Innate-Like B Lymphocytes

In addition to T cells, also the B cell compartment possesses innate members: B1 and MZ B lymphocytes, which are well characterized in the mouse model. As innate T lymphocytes, they exhibit a pre-programed phenotype allowing them to spontaneously secrete so-called natural antibodies (mostly IgM) and/or rapid proliferation and differentiation into antibody-secreting cells (also preferentially of the IgM isotype) upon stimulation with T cell-independent antigens. Whereas murine B1 lymphocytes locate essentially in peritoneal and pleural cavities, MZ B lymphocytes are found principally in the MZ of the spleen. These innate B lymphocytes have been the subject of recent reviews (15, 16), and we only briefly summarize some characteristics here in comparison with innate T lymphocytes. For example, it has been described that an invariant BCR of both B1 and MZ B lymphocytes can recognize lipids, both exogenous (phosphorylcholine attached to bacterial cell-wall glycolipid components) and endogenous (oxidized low-density lipoprotein and lipids exposed by apoptotic cells) (115). Thus, like iNKT cells, innate B lymphocytes can be activated by both bacterial-derived and endogenous lipids, but without the need of a presenting element. Although adult bone marrow is capable to generate these cells, this occurs at a lower efficiency compared to neonatal or fetal-derived stem cells and thus it seems that B1 lymphocytes are mainly generated before birth (15, 88, 116). In contrast, mouse MZ B lymphocytes are mainly generated after birth (16), thus again ranking subsets of innate lymphocytes according to their “innateness”: B1 lymphocytes are more innate than MZ B lymphocytes.

In the human system, it would be interesting to know whether these innate B lymphocytes, especially the most innate B1 cells, are reconstituted after HSCT. However, these investigations are complicated because of the lack on a consensus on the clear identification of B1 lymphocytes in human. Indeed, recently a population of cells have been described that show functional similar characteristics as murine B1 lymphocytes (117). However, the identification of these cells has generated controversy, including the finding that (part of) these cells might rather be pre-plasmablasts (118, 119). The candidate human B1 lymphocytes described by Griffin et al. (117) were present in cord blood and declined with age, indicating their preferential generation before birth as for mouse B1 lymphocytes, but this needs further investigation. In contrast to mouse MZ lymphocytes, it appears that human MZ B lymphocytes are mainly generated in the fetus (16, 120). A similar species difference exists for iNKT cells, which are also mainly generated after birth in mice, but before birth in human (Table 1). However, direct comparison between the mouse and human immune system in early life should be done with the notion that in general the human immune system develops relatively early during gestation compared to the development in mice. In experiments to investigate immune responses, one therefore usually uses day 7 old mice to compare immune responses with human neonates (121, 122).

## NK Cells and Other Innate Lymphoid Cells

To segregate them from T cells and B cells, lymphocytes without antigen receptor rearrangement are collectively termed ILCs (3). These ILCs include the well-established NK cells, lymphoid tissue inducer (LTi) cells (123), and a family of developmentally related ILCs that are effectors and regulators in immunity and in tissue development and remodeling (124).

Natural killer cells were originally described as a cell subset capable of rapidly eliminating target cells without the need for prior sensitization (125). NK cells are important in conferring protection from viral infections, such as CMV [see also “Immune Response to Cytomegalovirus in Early Life” within this Research Topic (Huygens et al. under review)], and in the immunosurveillance of transformed cells. NK cell activation is the result of the integration of signals provided by activation receptors such as NKG2D, recognizing stress-induced ligands such as MICA in human and Rae-1 in mice, and inhibitory receptors such as members of the killer-cell immunoglobulin-like receptor (KIR) family in humans and C-type lectin Ly49 family in mice, recognizing the presence of “self” by binding MHC class I molecules. Besides their role in balancing NK activity in the periphery, KIR and Ly49 members play also an important role in the “education” or “licensing” of NK cells during their development (126, 127). It has been shown recently that human fetal NK cells differentiate early *in utero* and are highly responsive to cytokines and antibody-mediated stimulation but respond poorly to MHC class I-negative target cells. Indeed, in contrast to adult NK cells, it appears that expression of KIRs did not educate fetal NK cells but rendered them hyporesponsive to target cells lacking MHC class I (128). This selective hyporesponsiveness could contribute to fetal–maternal tolerance *in utero*. Thus, human NK cells can be made early during gestation, but they appear to be functionally different than adult NK cells. It remains to be established whether this reflects different waves of NK cell development: an early wave of fetal NK cells and post-natal wave of NK cells. Upon HSCT in human, NK cells appear to reconstitute well and rapidly, with influence of CMV on their reconstitution [see also “Immune Response to Cytomegalovirus in Early Life” within this Research Topic (Huygens et al. under review)] (129), while other ILC populations appear to reconstitute more slowly (130).

In contrast to the KIR repertoire on human fetal NK cells, the Ly49 repertoire on mouse fetal and neonatal NK cells is very restricted: among the entire murine Ly49 family, Ly49E is the only member expressed before birth (131). Conversely, whereas the population of NK cells bearing one or more of the other Ly49 members progressively increases after birth, the percentage of Ly49E-expressing cells gradually decreases with almost no adult NK cells displaying this receptor (131). Fetal liver progenitor cells were shown to be much more efficient in the generation of Ly49E-expressing NK cells than adult bone-marrow progenitors (132). Interestingly, Ly49E is also the only Ly49 member expressed by Vγ5Vδ1 cells (DETC) (133). Despite this particular fetal expression pattern, it appears that Ly49E does not play a crucial role in differentiation and/or function of (fetal) NK cells (134).

Natural killer cells and other ILCs share common precursors during ontogeny as demonstrated in mouse models. Thereby, development of all sorts of ILCs depends on common gamma-chain cytokine signaling (135), in particular via IL-7R and IL-15R (3, 136), and the expression of the transcription factor helix–loop–helix inhibitor Id2 (137, 138). In addition, while the basic leucine zipper transcription factor E4BP4/Nfil3 was initially described as specifically required for the development and maturation of NK cells (139, 140), it is emerging very recently that E4BP4/Nfil3 is also crucial for the development of all other ILC subsets (141, 142). Also recently, two independent studies identified precursors that were specific for the ILC lineages. A common, IL-7R- and Id2-expressing progenitor named “CHILP” gives rise to all IL-7R-expressing, “helper-like” ILC lineages including LTi cells but lacks potential to differentiate into B and T cells or NK cells (143). In addition, a precursor termed ILCP, defined by transient expression of high amounts of the transcription factor PLZF, gives rise to all ILC lineages except LTi cells but lacks potential to differentiate into B and T cells or NK cells (144). Importantly, both CHILP and ILCP were found in fetal liver and adult bone marrow. Also, both precursors reconstituted ILC1, ILC2, and ILC3 lineages upon transfer into Rag- and IL-7R- deficient recipients (into *Rag2*-/-*Il2rg*-/- mice). Collectively, these transfer experiments advocated that ILC1, ILC2, and ILC3 populations can be regenerated from ILC precursors in an adult organism. However, it is currently unclear to what extent ILC precursors and ILCs are *de novo* regenerated in the steady state (Andreas Diefenbach, personal communication). Also, it is presently not clear how efficiently ILCs as well as CHILP and ILCP precursors are regenerated after HSCT. In human HSCT, current protocols to monitor immune recovery do not include ILCs (145). Moreover, recipient CD45^+^CD3^-^RORγt^+^ ILCs persisted long-term post-HSCT in the intestinal lamina propria of transplanted mice showing that ILCs are relatively radio-resistant (146). At the same time, more than 1/3 of the ILCs in recipient lamina propria were donor-derived 2 months post-HSCT, suggesting that it is likely that development of ILCs is not restricted to early ontogeny (146).

## Concluding Remarks and Perspectives

After reviewing the ontogeny of innate lymphocytes, it becomes clear that some subsets (such as mouse Vγ5Vδ1 T cells and B1 lymphocytes) are more innate than others (such as ILCs, MAIT, and MZ B lymphocytes). Overall, the preferential generation of innate lymphocyte subsets before birth appears to have implications for their reconstitution after HSCT, but this does not need necessarily to be the case, as indicated for human iNKT cells (Table 1). Many innate lymphocytes, in particular the very innate γδ T cell ones, expand in the periphery and persist as long-lived self-renewing cells. At the same time, these cells show above-average resistance to ionizing radiation (57–59, 147). This observation can be a confounding factor for determining the regeneration potential of innate lymphocyte populations.

A further common attribute of Vγ9Vδ2 T cells, iNKT cells, and MAIT cells is that all recognize small metabolites derived from host cellular or (commensal) microbial metabolism using semi-invariant TCRs and monomorphic presenting/selecting elements (Table 1). This contrasts with conventional or mainstream αβ T cells that use TCRs with highly variable CDR3 regions recognizing processed peptides from pathogen-derived proteins presented by polymorphic MHC molecules. These metabolites, together with their selecting/education element, can play an important role during the development of these innate cells. Recently, it has been shown that the TCR signaling of murine innate-like T lymphocytes, such as the IL-17-producing Vγ6Vδ1 subset, is critical for their development but that this signaling is attenuated in the periphery where these cells become more responsive to innate-like signals such as derived from cytokine receptors (69). However, since this did not apply to iNKT cells, it appears not to be a general phenomenon for all innate-like T lymphocytes. As discussed by Wencker et al. (69), by altering their TCR response mode, subsets of innate-like T cells may resemble ILC (that by definition lack constraint by a TCR). However, these innate-like T cells are still different from ILCs as they are not fully anergic to TCR signals and the prerequisite for developmental TCR signaling may provide a means of “quality control” for lymphocytes entering the innate compartment that is not available for ILCs.

The production of different types of innate lymphocytes before birth compared to later in life (such as expressing different TCRs as for γδ T cells, or showing a different “KIR educational response” for human NK cells), may be the result of a “layered” production of immune cells by fetal HSC versus adult HSCs. While evidence for this exists for some subsets, such as the fetal waves of murine Vγ5Vδ1 T lymphocytes (80) and B1 lymphocytes (15) with life-long self-renewal, this still needs to be established for others (including human Vγ9Vδ2 T cells and NK cells). Also for other cells of the immune system, a similar mechanism of fetal waves appears to be operational: in steady state, most tissue macrophage populations in mice are derived from embryonic precursors, are seeded before birth, and can maintain themselves in adults by self-renewal (148). Also, compared to adult HSC, human fetal HSC gives rise to a distinct type of conventional CD4 T cells with a bias toward immune tolerance (149).

Finally, it will be interesting to further investigate whether the “age” of human stem cells in stem cell transplantation (such as age of donors, or adult bone marrow/peripheral blood HSCT versus UCBT) can influence the type of innate lymphocytes that is reconstituted. This can have important clinical consequences such as influence on graft-versus-leukemia effect or post-transplantation CMV infection [e.g., Ref. (79, 86)].

## Conflict of Interest Statement

The authors declare that the research was conducted in the absence of any commercial or financial relationships that could be construed as a potential conflict of interest.
