# Corrigendum: Ontogeny of Innate T Lymphocytes – Some Innate Lymphocytes Are More Innate Than Others

**DOI:** 10.3389/fimmu.2015.00624

**Published:** 2015-12-14

**Authors:** David Vermijlen, Immo Prinz

**Affiliations:** ^1^Faculty of Pharmacy, Université Libre de Bruxelles (ULB), Bruxelles, Belgium; ^2^Institute of Immunology, Hannover Medical School, Hannover, Germany

**Keywords:** T cell, gammadelta T cells, ILC, neonatal, HSCT, BTN3A1, Skint1

We have found an error in our review article “Ontogeny of innate T lymphocytes – some innate lymphocytes are more innate than others.”

On page 6, under the section “Mucosa-associated invariant T cells”:

“In humans, this TCR consists of Vα7.2–Jα22….”

Instead of “Jα22,” this should be “Jα33.”

The same error is present in Table 1, under “MAIT – Human,” row “(Semi-)invariant TCR”: should be Jα33.

## Author Contributions

DV and IP co-wrote the original manuscript and corrigendum.

## Conflict of Interest Statement

The authors declare that the research was conducted in the absence of any commercial or financial relationships that could be construed as a potential conflict of interest.

